# Patients Prefer a Virtual Reality Approach Over a Similarly Performing Screen-Based Approach for Continuous Oculomotor-Based Screening of Glaucomatous and Neuro-Ophthalmological Visual Field Defects

**DOI:** 10.3389/fnins.2021.745355

**Published:** 2021-10-06

**Authors:** Rijul Saurabh Soans, Remco J. Renken, James John, Amit Bhongade, Dharam Raj, Rohit Saxena, Radhika Tandon, Tapan Kumar Gandhi, Frans W. Cornelissen

**Affiliations:** ^1^Department of Electrical Engineering, Indian Institute of Technology – Delhi, New Delhi, India; ^2^Laboratory of Experimental Ophthalmology, University Medical Center Groningen, University of Groningen, Groningen, Netherlands; ^3^Department of Biomedical Sciences of Cells and Systems, Cognitive Neuroscience Center, University Medical Center Groningen, University of Groningen, Groningen, Netherlands; ^4^Department of Ophthalmology, Dr. Rajendra Prasad Centre for Ophthalmic Sciences, All India Institute of Medical Sciences, New Delhi, India

**Keywords:** visual field defects, eye movements, virtual reality, cross-correlogram, perimetry, user experience, glaucoma, neuro-ophthalmology

## Abstract

Standard automated perimetry (SAP) is the gold standard for evaluating the presence of visual field defects (VFDs). Nevertheless, it has requirements such as prolonged attention, stable fixation, and a need for a motor response that limit application in various patient groups. Therefore, a novel approach using eye movements (EMs) – as a complementary technique to SAP – was developed and tested in clinical settings by our group. However, the original method uses a screen-based eye-tracker which still requires participants to keep their chin and head stable. Virtual reality (VR) has shown much promise in ophthalmic diagnostics – especially in terms of freedom of head movement and precise control over experimental settings, besides being portable. In this study, we set out to see if patients can be screened for VFDs based on their EM in a VR-based framework and if they are comparable to the screen-based eyetracker. Moreover, we wanted to know if this framework can provide an effective and enjoyable user experience (UX) compared to our previous approach and the conventional SAP. Therefore, we first modified our method and implemented it on a VR head-mounted device with built-in eye tracking. Subsequently, 15 controls naïve to SAP, 15 patients with a neuro-ophthalmological disorder, and 15 glaucoma patients performed three tasks in a counterbalanced manner: (1) a visual tracking task on the VR headset while their EM was recorded, (2) the preceding tracking task but on a conventional screen-based eye tracker, and (3) SAP. We then quantified the spatio-temporal properties (STP) of the EM of each group using a cross-correlogram analysis. Finally, we evaluated the human–computer interaction (HCI) aspects of the participants in the three methods using a user-experience questionnaire. We find that: (1) the VR framework can distinguish the participants according to their oculomotor characteristics; (2) the STP of the VR framework are similar to those from the screen-based eye tracker; and (3) participants from all the groups found the VR-screening test to be the most attractive. Thus, we conclude that the EM-based approach implemented in VR can be a user-friendly and portable companion to complement existing perimetric techniques in ophthalmic clinics.

## Introduction

The measurement of visual fields through standard automated perimetry (SAP) is the cornerstone of diagnosing and assessing ocular disorders. However, the current techniques in SAP have requirements that limit application in specific patient groups. Firstly, SAP requires the patient to click a button within a short time on perceiving the stimulus. Secondly, they have to keep their eyes fixated on a central cross while the testing light is projected onto different parts of their visual field. Thirdly, SAP is cognitively demanding – there is a learning curve associated with the test (Wall et al., 2009), leading to unreliable results within (due to fatigue) and across tests, besides increasing the overall test duration (Montolio et al., 2012). Consequently, not all patient groups can perform SAP easily – for example, the elderly with slower reaction times (Johnson et al., 1988; Gangeddula et al., 2017), children who have shorter attention spans (Lakowski and Aspinall, 1969; Kirwan and O’keefe, 2006; Allen et al., 2012), and patient groups with fixation disorders (Ishiyama et al., 2014; Hirasawa et al., 2018). These issues render SAP ineffective in such groups and interfere with diagnosing the ocular disorder. Therefore, our group developed and clinically tested a novel approach (Grillini et al., 2018; Soans et al., 2021) to screen for visual field defects (VFDs) based on eye movements (EMs).

The method is based on analyzing a patient’s EM while performing a very simple task: tracking a blob that moves and jumps on a screen. The intuition behind this task is as follows: smoothly following the moving blob will be harder for someone with a VFD because depending on the depth of the defect, the blob can temporarily become less visible or even invisible when it falls within a scotoma. As a result, the participant can no longer track the stimulus and will need to make additional EMs to find the target again. This will take more time and result in an increased delay and larger spatial errors. Central and peripheral defects differentially affect this. Using simulations (Grillini et al., 2018) and measurements in actual patients (Soans et al., 2021), we have shown that the method works very well (VFD simulations: Accuracy = 90%, TPR = 98%; Patients: Accuracy = 94.5%, TPR = 96%). However, our new method still has some aspects that may limit its use. It requires a large and expensive screen-based eye-tracker and still requires participants to keep their chin and head stable. Virtual reality (VR) holds promise to overcome some of these limitations. It allows for free head movements in all directions and has an increased field of view (FOV). Moreover, VR devices are portable, relatively cheap and allow for precise control over experimental conditions (Scarfe and Glennerster, 2019).

However, we do not know how well a VR-based version would work in terms of performance and if patients would appreciate using a VR device. Therefore, in this study, we explored if patients can be screened for VFDs based on their EM in a VR-based framework. Moreover, testing the human–computer interaction (HCI) aspects of this approach was another of our key goals. Consequently, we wanted to know if our VR-based framework can provide an effective and enjoyable user experience (UX) compared to our previous approach on the screen-based eye tracker and conventional SAP.

## Materials and Methods

### Participants

Fifteen patients with glaucoma, 17 patients with a neuro-ophthalmic disorder, and 21 controls volunteered to participate. All patients were recruited from the All India Institute of Medical Sciences – Delhi (AIIMS), New Delhi, India. The patients were required to have stable visual fields and have a best-corrected visual acuity (BCVA) of 6/36 or better. The control group was required to have a BCVA of 6/9 (0.67 or ≤0.17 logMAR) or better in both eyes. Participants with amblyopia, nystagmus, and strabismus were excluded from the present study. We also excluded participants with any conditions that affected the extraocular muscles as the limited range of their EMs could confound the results of our present paradigm. For the performance analysis, two participants with a neuro-ophthalmic disorder and six control participants were excluded because of poor eye-tracking data either in the VR or the screen-based eye tracker setup. However, all 53 participants were included in the UX analysis. Table 1 shows the demographics of the included participants for the performance analysis of the study. All participants gave their written informed consent before participation. The study was approved by the ethics board of AIIMS and the Indian Institute of Technology – Delhi (IITD). The study adhered to the tenets of the Declaration of Helsinki.

**TABLE 1:** Demographics and clinical characteristics of the participants.

Characteristics	Neuro-ophthalmological disorders (*N* = 15)	Glaucoma (*N* = 15)	Controls (*N* = 15)
Age (year)	31.8 (11.55)	40.33 (12.1)	32.93 (10.22)
Age range (year)	18–56	26–67	23–61
Male (sex)	9 (60%)	9 (60%)	11 (73.3%)
BCVA	0.78 (0.27)	0.89 (0.16)	1 (0)
BCVA range	0.16–1	0.67–1	1
Subtypes	Optic neuritis (*n* = 4)	PACG (*n* = 6)	
	Papilledema (*n* = 3)	NTG (*n* = 3)	
	Pituitary adenoma (*n* = 2)	POAG (*n* = 2)	
	IIH (*n* = 2)	Secondary glaucoma (*n* = 2)	
	TON (*n* = 1)	JOAG (*n* = 1)	
	Diffused astrocytoma (*n* = 1)	GON (*n* = 1)	
	Optic atrophy (*n* = 1)		
	LHON (*n* = 1)		

*The values are represented as mean (SD) or number (%). There were no statistically significant differences between the means of the three age groups as determined by one-way ANOVA [*F*(2,42) = 2.5128, *p* = 0.093]. IIH, idiopathic intracranial hypertension; TON, toxic optic neuropathy; LHON, Leber’s hereditary optic neuropathy; JOAG, Juvenile open angle glaucoma; GON, glaucomatous optic neuropathy.*

### Experimental Setups

The BCVA of the participants were first refracted using a Snellen chart with optimal correction for viewing distance. Table 1 shows the BCVA (in decimal units) for all three groups. Next, the participants were assigned to three different experimental setups in a counterbalanced manner. Below, we explain these setups in more detail.

#### Standard Automated Perimetry

A visual field assessment was performed for each eye (monocularly) on the Humphrey Field Analyzer (HFA) 3 – Model 860 (Carl Zeiss Meditec, Jena, Germany). A Goldmann III stimulus and the Swedish Interactive Threshold Algorithm Fast (SITA-Fast) was used in the assessment. Moreover, we used the 30-2 grid, i.e., a large FOV, to better evaluate and characterize the VFD. The different types of VFD observed and evaluated by SAP in the neuro-ophthalmic and glaucoma patient groups can be seen in Supplementary Tables 1, 2. The severity of the VFD was categorized according to the Hodapp–Parrish–Anderson classification (Hodapp et al., 1993) of the worse eye. The neuro-ophthalmic disorder group had six early-stage, four moderate, two advanced and three severe patients. The glaucoma group had five early-stage, eight moderate, one advanced, and one severe patient.

#### The Screen-Based Eye Tracker Setup

Here, we use the Tobii T120 (Tobii Technology, Stockholm, Sweden) screen-based eye tracker (size: 33.5 cm × 28 cm). The gaze positions were acquired at a sampling frequency of 120 Hz, downsampled to 60 Hz to match the refresh rate of the built-in screen. A 5-point custom-made calibration routine was performed before each experimental session. A session would be allowed to begin if the average error of the calibration was within 1° of visual angle as long as the maximum error was below 2.5°. A chin-rest was placed at a distance of 60 cm from the eye tracker to minimize head movements. We also used a Tobii infrared-transparent occluder so that the task could be done monocularly without hindering the eyetracker’s ability to monitor the gaze positions. The stimulus and experiment were designed with custom made scripts in MATLAB R2018b using the Psychtoolbox (Brainard, 1997; Pelli, 1997) and the Tobii Pro Software Development Kit (SDK) (Tobii, Stockholm, Sweden).

#### The Virtual Reality Setup

The FOVE0 (Fove Inc., Tokyo, Japan) VR head-mounted device (HMD) was used, which has a resolution of 2560 × 1440 pixels split across the two eyes. The HMD also has built-in eye-tracking capabilities – a stereo infra-red system with a tracking accuracy of less than 1° of visual angle error. The gaze positions were acquired at 120 Hz, downsampled to 70 Hz to match the refresh rate of the HMD screen. The FOVE does not include optical correction at present, but it can perform six-DOF head position and orientation tracking. However, we disabled its rendering such that the participant is afforded an experience similar to that of placing their forehead on a chin-rest. A “smooth pursuit” calibration procedure provided by FOVE wherein the participant has to look at a green dot moving in a circle was performed before the start of each experimental session. The calibration was deemed to be successful if the average error was within 1.15° of visual angle. The VR scene consisted of two virtual cameras placed at a distance of 6.4 cm (equivalent to interocular distance), and the virtual screen size was 100 cm × 60 cm. Either of the virtual cameras could be disabled so that the task could be done monocularly. The device was connected to a laptop with the following specifications: Dell Alienware m15-R3 with Nvidia RTX 2070 graphics card (8 GB video memory), 16 GB RAM, Intel Core i7-10750H 2.5 GHz processor, and Windows 10–64 bit operating system. The scripts to create the VR environment, render the tasks, and collect the eye-tracking data were programmed through C#, Unity 2017, and the FOVE SDK.

### The Stimulus and Tracking Task

The stimulus in both the VR and screen-based eye-tracking setups consisted of a Gaussian luminance blob of 0.43° placed at a virtual distance of 135 cm (from the center of the two cameras in VR) and 60 cm (by the chin-rest for the screen-based eye tracker) – so as to correspond to the Goldmann size III stimulus. The blob had a luminance of ∼165 cd/m^2^ on a uniform gray background (∼150 cd/m^2^). The blob moved according to a Gaussian random walk in two different modes: (1) a “smooth” mode where the blob moved continuously in the random walk or (2) a “displaced” mode where the blob made a sudden jump to a new location in the screen every 2 s. The participants were asked to follow the moving blob and were told to blink naturally as and when required. Each trial lasted for 20 s each, and there were six such trials, amounting to a total test time of about 5 min, including calibration and resting breaks.

### Eye-Tracking Data Preprocessing and Spatio-Temporal Properties

In both the VR and screen-based eyetracker, we first obtain the gaze positions in terms of screen coordinates which is then converted into visual field coordinates. Subsequently, the gaze data is corrected for blinks according to a custom algorithm (Soans et al., 2021; see Supplementary Material for description). Owing to differences in the sampling rate and the physical screen size of the devices, we use empirically determined spike thresholds of 60°/s and 190°/s in the vertical gaze velocity components of the FOVE and the Tobii gaze data, respectively. A particular trial is excluded if more than 33% of its total duration consists of data loss by either blinks or missing data.

Once we have the blink-filtered eye-tracking data, we quantify the spatio-temporal properties (STP) of EM using the eye movement correlogram (EMC) analysis (Mulligan et al., 2013). The EMC is an analytical technique that can be used to quantify both temporal and spatial relationships between the time series of a set of stimuli and their corresponding responses. The analysis provides three types of STP:

(1) Temporal properties (Bonnen et al., 2015): These include cross-correlograms (CCGs) that yield four properties (*CCG Amplitude*, *CCG Mean*, *CCG Standard Deviation*, and *CCG variance explained*) for the horizontal and vertical components of the eye positions, respectively. These properties are reflective of temporal correlation between the stimuli and response, smooth pursuit latency, temporal uncertainty, and resemblance of the tracking performance to a Gaussian distribution, respectively.
(2) Spatial properties (Grillini et al., 2018; Soans et al., 2021): These include positional error distributions (PEDs) that yield additional four properties (*PED Amplitude*, *PED Mean*, *PED Standard Deviation*, and *PED variance explained*) for the horizontal and vertical components of the eye positions, respectively. These properties indicate the most frequently observed positional error, spatial offset, spatial uncertainty, and the resemblance of the PED to a Gaussian distribution, respectively.
(3) Integrated properties (Grillini et al., 2018; Soans et al., 2021): These two properties (*Cosine similarity* and *Observation noise variance*) incorporate both the spatial deviations and the temporal delays in the stimulus and response time series.

A full description of the STP, including the range of values each can take, is provided in Supplementary Table 3. In total, we end up with 80 features for each participant [10 STP × 2 modes (smooth and displaced) × 2 components (horizontal and vertical) × 2 eyes].

### Spatio-Temporal Properties Analysis

Our goal here was twofold: (1) to see if the VR framework could distinguish between the three clinical groups and (2) if the STP obtained in the VR framework was similar to those obtained from the screen-based eye tracker. To this end, we first performed a principal component analysis (PCA) on the centered and scaled 80 features of every participant in both the frameworks to remove redundant features. Next, we retained the components that together have an explained variance of at least 95%. Then, we performed *k*-means clustering to identify the different clusters pertaining to the clinical groups. After that, we used the Silhouette criterion (Rousseeuw, 1987) to choose the optimum number of clusters. Finally, to compare the STPs obtained from the VR framework and those from the screen-based eye tracker, we first visualized the correlations between three key STPs (Soans et al., 2021) using scatter plots. Subsequently, we computed the pairwise correlation coefficients between each pair of the 80 STPs in the FOVE and Tobii gaze data. We then report on the number of STPs that had significant correlation between the two devices.

### User-Experience Questionnaire

#### Statements and Dimensions

Our second goal was to test out the HCI aspects of the three setups. Therefore, we prepared a User Experience Questionnaire (UEQ) that incorporated six dimensions, namely: *Competence*, *Perspicuity*, *Immersion*, *Comfort*, *Aesthetics*, and *Attractiveness*. These dimensions were borrowed and adapted to our study from the standard UEQ (Schrepp et al., 2014) and the Game Experience Questionnaire (GEQ) (IJsselsteijn et al., 2013). In the *Competence* dimension, the statement was: “I found the test easy to perform.” For the *Perspicuity* dimension – which evaluates the clarity of the test instructions, the statement was: “I understood the test instructions clearly.” Likewise, the *Immersion* dimension evaluates the sensory and cognitive load of the participant with the statement: “It was easy to focus on the given task.” In the *Comfort* dimension, the statement was designed to evaluate post-test fatigue with: “I felt comfortable during the task (physical or otherwise).” In the *Aesthetics* dimension, the statement was: “Overall, I found the test enjoyable.” Finally, the *Attractiveness* dimension evaluated the overall impression of the test setup with the statement: “Please rank the tests from most attractive to least attractive.” A true-translation copy of the questionnaire in Hindi – the local language in New Delhi – was also provided to the participant when required. Table 2 shows the English questionnaire.

**TABLE 2:** The UEQ statements for the three setups.

Instruction – for each question, please tick the relevant box		Strongly disagree (1)	Disagree (2)	Neutral (3)	Agree (4)	Strongly agree (5)
1.	I found the test easy to perform: 1) SAP – HFA 2) EMC – eyetracker – Tobii 3) EMC – virtual reality – FOVE	□ □ □	□ □ □	□ □ □	□ □ □	□ □ □
2.	I understood the test instructions clearly: 1) SAP – HFA 2) EMC – eyetracker – Tobii 3) EMC – virtual reality – FOVE	□ □ □	□ □ □	□ □ □	□ □ □	□ □ □
3.	It was easy to focus on the given task: 1) SAP – HFA 2) EMC – eyetracker – Tobii 3) EMC – virtual reality – FOVE	□ □ □	□ □ □	□ □ □	□ □ □	□ □ □
4.	I felt comfortable during the task (physical or otherwise): 1) SAP – HFA 2) EMC – eyetracker – Tobii 3) EMC – virtual reality – FOVE	□ □ □	□ □ □	□ □ □	□ □ □	□ □ □
5.	Overall, I found the test enjoyable: 1) SAP – HFA 2) EMC – eyetracker – Tobii 3) EMC – virtual reality – FOVE	□ □ □	□ □ □	□ □ □	□ □ □	□ □ □
6.	Please rank the tests from most attractive to least attractive (most attractive – 1, attractive – 2, least attractive – 3)	SAP-HFA		EMC-Tobii		EMC-FOVE

*For questions 1–5, participants had to tick the appropriate box, while for the last question, they had to rank the setups.*

*SAP, standard automated perimetry; HFA, Humphrey Field Analyzer; EMC, eye movement correlogram.*

#### Scales and Statistical Analysis

The first five dimensions of our UEQ were rated on a 5-point Likert scale of 1 (strongly disagree), 2 (disagree), 3 (neutral), 4 (agree), and 5 (strongly agree). The participants’ response in these dimensions across the three setups was evaluated using a non-parametric Friedman’s test. The exact method was used to compute the *p*-values, and Kendall’s coefficient of concordance (*W*) was used to estimate the effect sizes. Cohen’s guidelines were used for the interpretation of the effect sizes (<0.2: no effect, 0.2–0.5: small-to-medium effect, 0.5–0.8: medium-to-large effect, and >0.8: large effect) (Cohen, 2013). *Post hoc* analyses using Wilcoxon signed-rank tests were conducted to compare two modalities directly (see section “Discussion” and Supplementary Table 4). These *post hoc* tests were corrected for multiple comparisons through Bonferroni’s correction for each dimension. The statistical analyses were done using SPSS (Version 26.0; Armonk, NY, United States: IBM Corp.). For the sixth dimension, participants had to rank the three tests from 1 (most attractive), 2 (attractive), and 3 (least attractive). The results for this dimension are described as stacked bar plots.

## Results

To summarize the results, the VR framework shows that neuro-ophthalmic patients had the highest average smooth pursuit latency compared to the glaucoma patients and controls. Moreover, these patients showed higher spatial and temporal uncertainties in both the “smooth” and “displaced” modes of the experiment. The framework also shows that glaucoma patients had a higher average latency than the other groups in the “displaced” mode of the experiment. Furthermore, the VR framework is able to distinguish the three participant groups based on the STP of their EM. Finally, participants from all groups rated the VR setup significantly higher than the screen-based eye tracker and SAP across the UX dimensions. We describe these results in more detail below.

### Spatio-Temporal Properties of Eye Movement in the Three Groups

**FIGURE 1:**
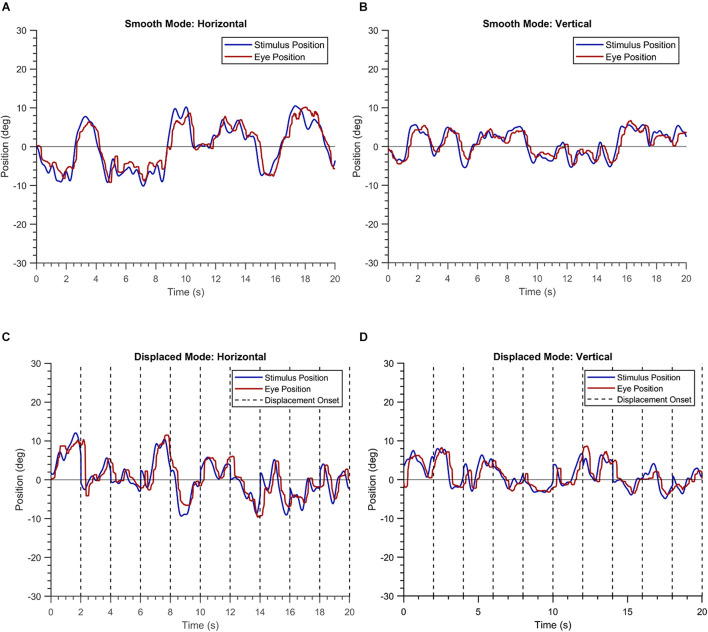
Plots of a single trial of a healthy participant in the “smooth” **(A,B)** and the “displaced” mode **(C,D)**.

The demographic information of the three groups is summarized in Table 1. Figure 1 shows the EM recorded for a single trial by the VR device in a healthy participant. The blob positions are shown in blue, while the gaze positions are shown in red. The left panel of the figure depicts the horizontal positional components of the blob and gaze positions, while the right panel depicts the corresponding vertical components. The overlap between the two time-series signals conveys that the participant is able to follow the moving blob in both modes of the experiment with ease.

**FIGURE 2:**
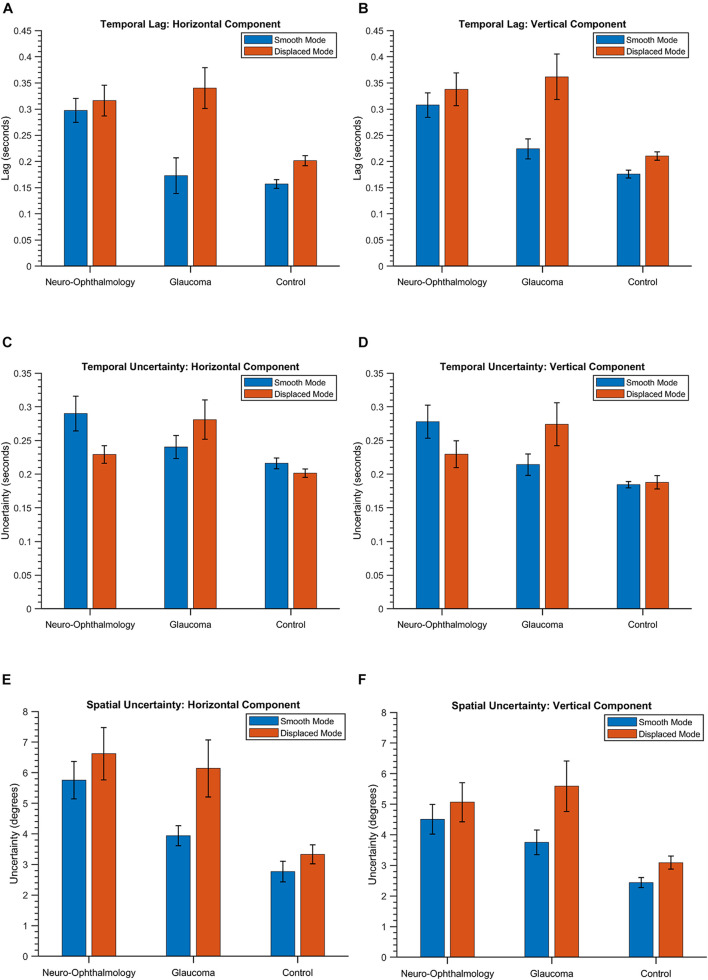
The group means and the corresponding 95% confidence intervals of the temporal lag **(A,B)**, temporal uncertainty **(C,D)** and spatial uncertainty **(E,F)** in both the modes and across the two positional components for the three groups. The values are obtained from the STPs *CCG Mean*, *CCG Standard Deviation*, and *PED Standard Deviation*, respectively (see section “Eye-Tracking Data Preprocessing and Spatio-Temporal Properties” and Supplementary Table 3).

First, we describe the results of three key STPs (Soans et al., 2021), i.e., temporal lag, temporal uncertainty, and spatial uncertainty in EM made by the three clinical groups in the VR device. Figure 2 shows the group means and the 95% confidence intervals for these features. These aspects are captured by the temporal features *CCG Mean*, *CCG Standard Deviation*, and the spatial feature *PED Standard Deviation*, respectively, in our STP feature list. Statistically significant differences were observed between the means of temporal lag for the three clinical groups as determined by a one-way MANOVA [*F*(8,78) = 22.05, *p* < 0.05; Wilk’s Λ = 0.09, partial η^2^ = 0.69]. *Post hoc* analyses using Tukey’s HSD test were conducted to compare the means of temporal lag between the clinical groups. Here, we observe that when the luminance blob moved smoothly, the neuro-ophthalmic patients had the highest temporal lag (Mean: [0.29, 0.31] seconds; *p* < 0.05) across the horizontal and vertical gaze components, respectively. The glaucoma patients had a temporal lag much lower than the neuro-ophthalmic group (Mean: [0.17, 0.23] seconds; *p* < 0.05) but similar to controls (Mean: [0.15, 0.18] seconds; glaucoma vs. controls, *p* = 0.57). In contrast, in the “displaced mode,” i.e., when the blob jumped every 2 s, the glaucoma group had the highest lag of all the groups (Mean: [0.34, 0.36] seconds) as compared to the neuro-ophthalmic patients (Mean: [0.31, 0.34] seconds) and controls (Mean: [0.20, 0.21] seconds; *p* < 0.05). However, no statistically significant difference (*p* = 0.42) was observed between the means of temporal lag for the glaucoma and the neuro-ophthalmic patient group in the “displaced” mode.

In terms of temporal uncertainty, statistically significant differences were observed between the means of the three groups {one-way MANOVA [*F*(8,78) = 15.06, *p* < 0.05; Wilk’s Λ = 0.15, partial η^2^ = 0.6]}. For the “smooth mode,” the neuro-ophthalmic group had again the highest uncertainty (Mean: [0.29, 0.28] seconds). However, the uncertainty in the glaucoma group (Mean: [0.24, 0.21] seconds) was no longer similar to the controls (Mean: [0.21, 0.18] seconds). In the “displaced” mode, the glaucoma group continues to have the highest uncertainty (Mean: [0.28, 0.27] seconds) followed by the neuro-ophthalmic group (Mean: [0.23, 0.23] seconds) and controls (Mean: [0.20, 0.19] seconds). All the differences between the mean temporal uncertainties of the three groups in the *post hoc* analyses (Tukey’s HSD) were statistically significant except for: (1) the horizontal gaze components of the control vs. glaucoma groups in the “smooth” mode (*p* = 0.12) and (2) the horizontal gaze components of the control vs. neuro-ophthalmic groups in the “displaced” mode (*p* = 0.07).

The three clinical groups behave differently in terms of spatial uncertainty. Statistically significant differences were observed between the mean values of the spatial uncertainties in the three groups {one-way MANOVA [*F*(8,78) = 15.62, *p* < 0.05; Wilk’s Λ = 0.14, partial η^2^ = 0.61]}. The neuro-ophthalmic group has a significant overlap between its values in both the modes, i.e., “smooth” mode – (Mean: [5.76°, 4.51°] of deviation in visual angle) and “displaced” mode – (Mean: [6.62°, 5.07°]). On the other hand, the uncertainties of the glaucoma group overlap with those of the neuro-ophthalmic group in the “displaced mode” – (Mean: [6.14°, 5.59°]) but are distinct from the latter in the “smooth” mode (Mean: [3.93°, 3.75°]). Finally, the controls have the lowest spatial uncertainty in both the “smooth” (Mean: [2.77°, 2.44°]) and the “displaced” modes (Mean: [3.33°, 3.09°]). All the mean values of spatial uncertainty between the three groups in the *post hoc* analyses (Tukey’s HSD) were statistically significant except for the horizontal and vertical gaze components of the neuro-ophthalmic vs. glaucoma groups in the “displaced” mode (*p* = 0.6 and *p* = 0.4), respectively. For a detailed comparison of these features (including the 95% confidence intervals), see Supplementary Table 5).

### Separation of the Clinical Groups Based on Spatio-Temporal Properties

**FIGURE 3:**
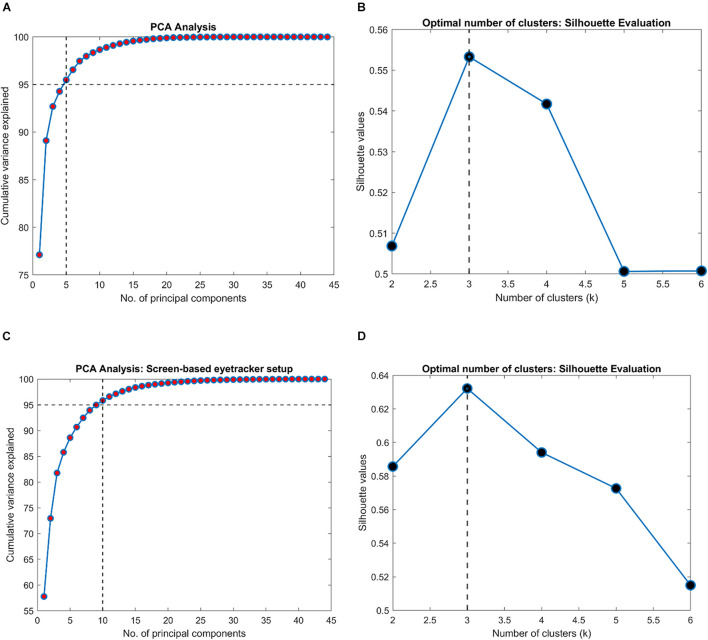
Results of clustering analysis in the VR and the screen-based eye tracker setup. **(A,C)** PCA analysis on the VR and the screen-based eye tracker gaze data indicating the number of components that together explained at least 95% of the variance in the STP features, respectively. A *k*-means clustering was then performed on this PCA-reduced data. **(B,D)** Evaluation of the optimal number of clusters for the VR and screen-based eye tracker setup using the Silhouette technique.

Next, we were interested in knowing if the VR framework could separate the clinical groups of individual patients based on their EM. Therefore, a PCA was performed on the 80 STP of every subject. The data was centered, standardized, and the STP were weighted by the inverse of their sample variance to account for different ranges (see Supplementary Table 3 for individual ranges). Figure 3 shows the results. The analysis revealed that five components together explained at least 95% of the variance (see Figure 3A). These five components were then used for subsequent *k*-means clustering. The optimal number of clusters to represent the data using the Silhouette evaluation was found to be three (Figure 3B) – equal to the number of clinical groups. The results of *k*-means clustering for the VR setup is shown in Table 3.

**TABLE 3:** Confusion matrix for the clustering analysis on the gaze data from the VR device.

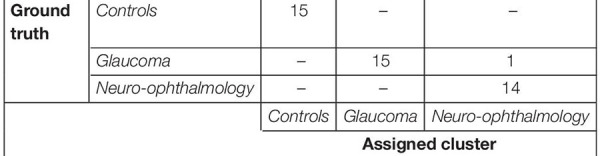

*The clinical data is used for comparison with ground truth. In the control group, all participants were correctly clustered. Among the patients, the neuro-ophthalmic group had a single false positive, which actually belonged to the glaucoma group.*

### Comparison of the Virtual Reality Framework to Screen-Based Eye Tracker in Terms of Spatio-Temporal Properties

Subsequently, we were interested to know whether the separation of the clinical groups in terms of STP by the VR framework was similar to that by the Tobii eye tracker. Therefore, we repeated the steps outlined in the preceding section on the gaze data of the screen-based eye tracker as well. The result of *k*-means clustering on the gaze data of the Tobii eye tracker is shown in Table 4. Here, 10 components were sufficient to explain 95% of the variance in the data, and the Silhouette evaluation resulted in choosing three as the number of optimal clusters (see Figures 3C,D).

**TABLE 4:** Confusion matrix for the clustering analysis on the gaze data from the screen-based eye tracker.

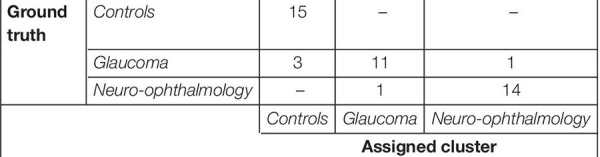

*All the controls were correctly clustered. However, three of the glaucoma subjects were assigned to the Control cluster, and one subject, each from the glaucoma and neuro-ophthalmic group, was misclassified to the other group, respectively.*

Tables 3, 4 show the confusion matrices for the *k*-means clustering results for the VR-based and the screen-based eye-tracker gaze data, respectively. All controls were correctly classified in the VR framework, and the clustering resulted in a single false positive for the neuro-ophthalmic group. The groupings based on the screen-based eye tracking, however, was much less coherent. Although all controls were still correctly classified, the control and the neuro-ophthalmic group overlapped with three participants and a single participant from the glaucoma group, respectively.

**FIGURE 4:**
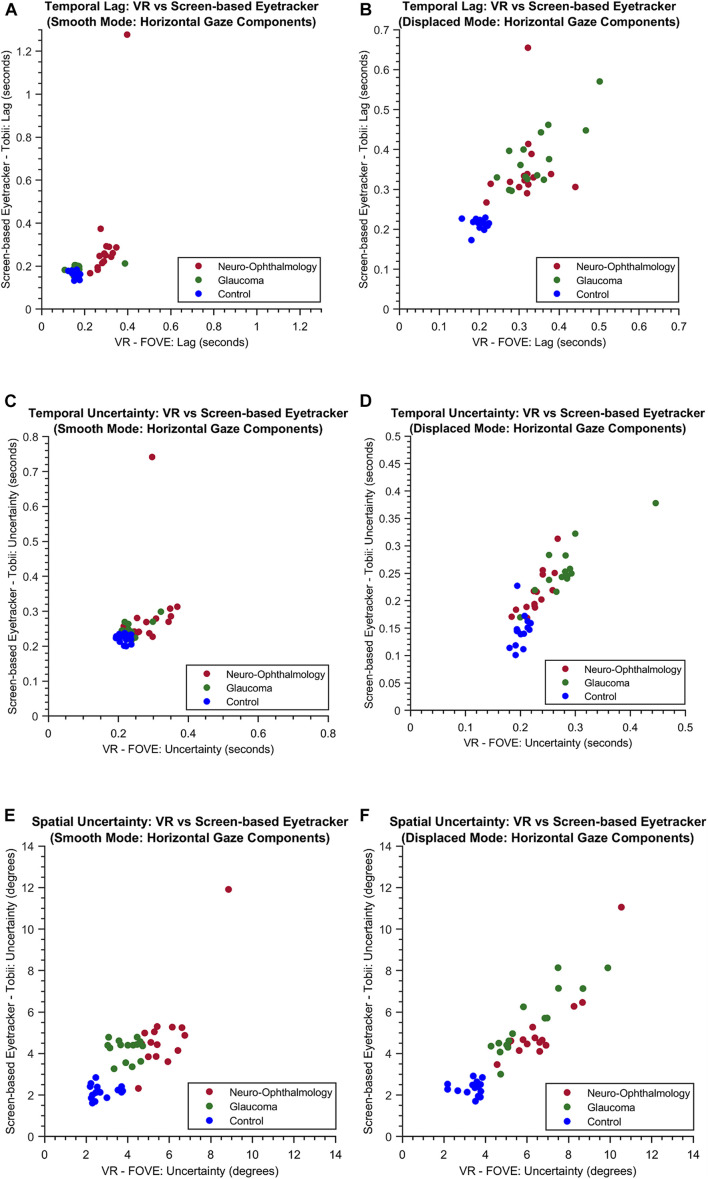
The scatter plots of the temporal lag **(A,B)**, temporal uncertainty **(C,D)** and spatial uncertainty **(E,F)** features obtained for both the experimental modes through VR and the screen-based eye tracker. For simplicity, only the horizontal components are shown.

Following this, we wanted to test the stability of STPs in both modalities. Therefore, we first visualized the relationship between three key STPs: Temporal Lag, Temporal Uncertainty, and Spatial Uncertainty derived from participants’ gaze data in the VR framework and the screen-based eye tracker. Figure 4 shows the scatter plots for these STPs in both the “smooth” and the “displaced” modes of the visual tracking task.

**FIGURE 5:**
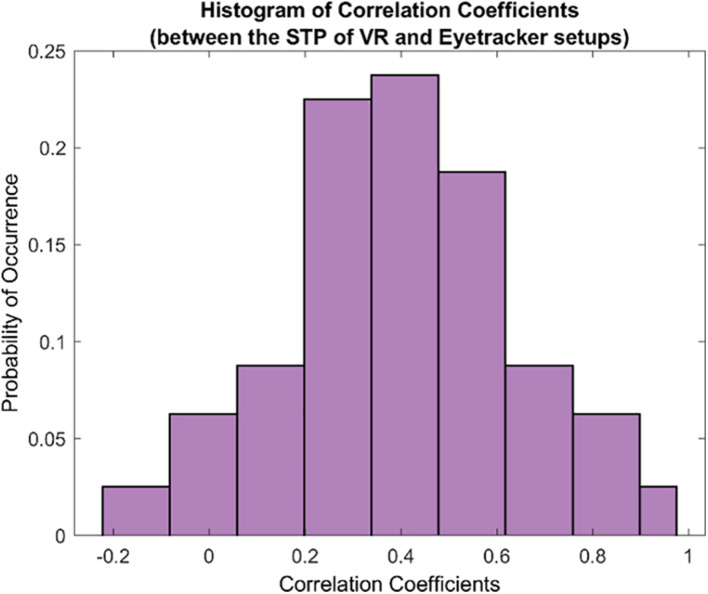
The histogram of correlation coefficients between the 80 STPs of the VR and the eye tracker setups. The unimodal histogram is centered around 0.4 ± 0.24. Fifty-three of the total 80 STPs were found to be significantly correlated across the two setups (see Supplementary Table 6).

Subsequently, we examined the overall relationship between the STPs in both the setups by computing the pairwise correlation coefficients between each pair of the 80 STPs in the FOVE and Tobii gaze data. Figure 5 shows the distribution of the pairwise correlation coefficients of the 80 STPs across the two setups. The histogram is unimodal and centered around a “moderately” positive correlation coefficient of 0.4 ± 0.24. Fifty-three of these 80 STPs (about 67%) were found to be significantly correlated across the setups (H_0_: the pairwise correlation coefficient is not significantly different from zero; rejected at *p* < 0.05). These significant STPs are listed in Supplementary Table 6.

### Evaluation of the User Experience Questionnaire

**FIGURE 6:**
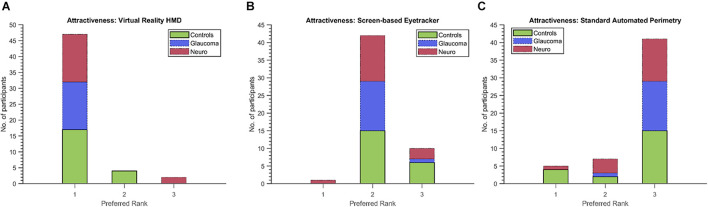
Preferred ranks for the three modalities (**A**: VR, **B**: Screen-based eyetracker, **C**: SAP) in the Attractiveness dimension of the UEQ. Forty-seven (including 17/21 controls, 15/15 glaucoma patients and 15/17 neuro-ophthalmic patients) of the total 53 participants rated the VR setup as the most attractive compared to only 5 (four controls and a neuro-ophthalmic patient) for the conventional SAP.

Finally, we used the non-parametric Friedman’s test to evaluate the ratings provided by participants from each group and for the first five dimensions of the UEQ. Table 5 shows the results. Statistically significant results were observed for the scores of these dimensions and in each participant group except for *Perspicuity* (all groups) and *Comfort* (neuro-ophthalmic; *p* = 0.309). *Post hoc* analyses using the Wilcoxon signed-rank test were conducted to compare two modalities within a participant group and a UEQ dimension (see section “Discussion” and Supplementary Material). These tests were corrected for multiple comparisons through Bonferroni’s correction. Still, we note that the statistical results of these additional comparisons could possibly be affected by circularity in the *post hoc* analyses (Kriegeskorte et al., 2009). Figure 6 shows the stacked plots for the sixth dimension – *Attractiveness*. Of the total 53 participants, 47 found the VR setup to be the most attractive of the three modalities. The screen-based eye tracker was judged to be the second most attractive, with 42 participants ranking it second and 10 ranking it third. The conventional SAP was found to be the least attractive, with only 5 participants ranking it as their preferred choice, 7 as second best, and 41 of them relegating it to be the least preferred of the screening modalities.

**TABLE 5:** The mean scores, results of Friedman’s test and the corresponding effect sizes on the ratings provided by the participants in each clinical group for each modality and the first five dimensions of the UEQ.

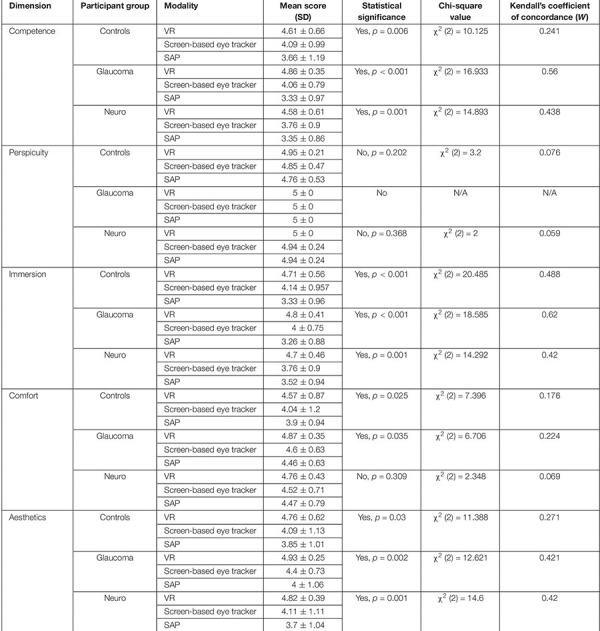

## Discussion

The main findings of the present study are that: (1) A VR device with built-in eye tracking is able to identify and distinguish patients with glaucomatous and neuro-ophthalmic VFD from controls based on their oculomotor characteristics. (2) The STPs obtained through our method is similar across the screen-based eye tracker and the VR setups, with the latter performing better. (3) Participants in each group prefer the VR setup in almost all the dimensions of UX. This would result in a quick, enjoyable, and effective way to screen for VFDs. Below, we discuss these findings in more detail.

### The Virtual Reality Framework Is Able to Identify Clinical Groups

Our primary objective was to explore if our new method deployed in a VR device could be used as an effective screening tool that can be applied to a fairly heterogeneous set of patients. Therefore, our cohort of participants included variegated types of VFDs within the neuro-ophthalmic and glaucoma group (see Supplementary Tables 1, 2). Despite this variance, the VR framework was successfully able to capture the oculomotor characteristics of the patients. The rationale behind the framework is simple – the presence of a VFD decreases a participant’s visual sensitivity, which in turn affects their pursuit performance. Moreover, if the participant cannot track a stimulus due to a VFD impairing their view of it, they must search for it, resulting in increased spatial errors. Together, these aspects alter the spatial and temporal parameters of the STP.

Figure 2 sums up the key oculomotor characteristics of the participant groups. The neuro-ophthalmic group exhibit slow performance during smooth pursuit (mean temporal lag is the highest – Figures 2A,B) and are rather inconsistent in their ability to track the smoothly moving stimulus [both the mean temporal and spatial uncertainty values are high – Figures 2C–F)]. This behavior agrees with the literature, with reduced visual sensitivity, increased response times and a limited “useful field of view” being observed in response to random transient signals in a group of hemianopic patients (Rizzo and Robin, 1996). Moreover, patients with cerebral lesions are known to have deficits in smooth pursuit as the target stimuli move toward the site of lesion (Thurston et al., 1988; Heide et al., 1996). This pattern of higher temporal lag and uncertainty continues in the “displaced” mode as well, i.e., when the luminance blob moved to a random location every 2 s. However, there is an intriguing pattern within the neuro-ophthalmic group, i.e., their temporal uncertainties in the “displaced” mode are lower than those in the “smooth” mode even though one would expect the opposite. A predictive visual search strategy by the participants may have contributed to this result. Meienberg et al. (1981) showed that hemianopic patients in a visual search task (over a period of repeated trials) start expecting that the target will appear in random locations. Consequently, they adopt a strategy of making a series of small saccades followed by an overshoot until the target is found. We also observed this pattern visually (see Supplementary Figure 1 for an example) but could not quantify the associated saccade dynamics owing to the relatively lower sampling frequencies of gaze data in both frameworks. Nevertheless, our group of neuro-ophthalmic patients had higher temporal lags and spatio-temporal latencies than controls (see Figures 2A,B). Numerous studies have shown that patients with neuro-ophthalmic VFD have prolonged latencies compared to controls in response to moving or stationary targets – often in the range of 20–100 ms more than controls (Sharpe et al., 1979; Meienberg et al., 1981; Traccis et al., 1991; Rizzo and Robin, 1996; Barton and Sharpe, 1998; Fayel et al., 2014). Our group has previously shown through simulations (Grillini et al., 2018; Gestefeld et al., 2020) and in actual patients (Soans et al., 2021) that this is indeed the case.

For the glaucoma group, the VR framework brings out some interesting oculomotor characteristics as well. When the luminance target stimulus moved smoothly, the glaucoma patients, while slower than controls, were much quicker than the neuro-ophthalmic group (see Figures 2A,B). This is because patients having glaucomatous VFD can usually perform smooth pursuit as their foveal vision is primarily intact. However, a closer look at Figures 2A–D reveals an interesting finding. While the glaucoma group had relatively low values for the temporal lag property in the “smooth” mode, their temporal uncertainty increases significantly (the error bars for glaucoma and the control group do not overlap in Figures 2C,D). This could be due to patients requiring integration on large spatio-temporal scales in addition to the decreased motion sensitivity typically observed in glaucoma (Shabana et al., 2003; Lamirel et al., 2014), which ultimately would lead to longer integration times in tracking the stimulus. In the “displaced mode,” the interpretations of our results are rather straightforward, with higher lags and spatio-temporal uncertainties being observed. This is because glaucoma patients exhibit peripheral visual field loss (PVFL) and are expected to make spatial errors and take more time as they make saccades to keep track of the randomly jumping blob. Although patients with PVFL do not show systematic changes in the duration of saccades and fixations compared to controls in visual search tasks that involve stationary targets (Luo et al., 2008; Wiecek et al., 2012), the EM behavior is different in the context of dynamic scenes. Studies involving dynamic movies of road traffic scenes showed that glaucoma patients made more saccades and fixations than controls (Crabb et al., 2010) and that it is possible to differentiate people with glaucomatous VFD from those with no VFD (Crabb et al., 2014). Moreover, glaucoma patients with different severity of VFD (early, moderate, and advanced) were observed to have a lower mean saccade velocity (Najjar et al., 2017) and significant delays in saccadic EMs when targets were presented in the peripheral regions (Kanjee et al., 2012).

### The Virtual Reality Framework Potentially Performs Better Than the Screen-Based Eye Tracker in Distinguishing Patient Groups

After identifying the patients’ oculomotor characteristics, a natural line of thought would be to see if the VR framework can classify them into one of the clinical groups. However, the number of participants in our study was relatively small (for machine-learning approaches). Therefore, we decided to use an unsupervised clustering technique, i.e., *k*-means clustering, even though we could label participants based on clinical investigations. Moreover, our aim at present was to see if the VR framework was able to at least distinguish between the different participant groups. Another concern when we started exploring the VR device as a potential tool for screening VFD was the fact that the FOVE had lower eye-tracking accuracy (Error <1° of visual angle) as compared to the Tobii T120 (Error <0.5° of visual angle). Despite leaps and bounds being made in HMD eye tracking technology (Clay et al., 2019), they are understandably inferior in terms of tech specifications to research-grade eye trackers. However, our choice of HMD for the VR framework eventually proved to be reasonable – Stein et al. (2021) showed that the FOVE had the lowest latency among a group of HMDs with built-in eye tracking.

In fact, we find that the key STPs across the two modalities are fairly stable (see scatter plots in Figure 4), which prompted us to investigate further the relationship between the entire set of STPs in the two setups. This is also encouraging considering the fact that participants viewed two different sets of random walks in the two setups. Moreover, the Silhouette evaluation returning the optimal number clusters to be 3 in both modalities confirms that there were indeed three separate groups of participants. These observations show that the VR device is comparable to the screen-based eye tracker in terms of capturing the STP of EM in the clinical groups.

Furthermore, our results show that the VR framework outperforms the screen-based eye tracker. Remarkably, there was only a single misclassification at the end of the clustering analysis in the VR approach compared to five in the eye tracker (see Tables 3, 4). This is despite the fact that the entire FOV of the VR device was not used to present stimuli (to keep a fair comparison with the limited FOV of the screen-based eye tracker – see section “Limitations and Future Directions”). Our results also indicate that the STPs of EM in the screen-based eye tracker were on the noisier side. The PCA analysis required 10 components to explain 95% variance in this data compared to only 5 for the VR framework (see Figures 3A,C). We think that this additional noise may be due to the Tobii infrared-transparent occluder as it acts as an additional physical barrier between the screen of the eyetracker and the participant. Moreover, we had noticed that calibration in the screen-based eye tracker sometimes took longer due to the positioning of the occluder.

### Virtual Reality Is Preferred Across All Aspects of the User Experience Questionnaire

Despite SAP techniques possessing advantages such as a wealth of normative data, testing multiple field locations with different luminance thresholds, they are still very much operator dependent. Moreover, they are expensive and bulky – often limited to major ophthalmic and tertiary care centers. These limitations of SAP have been studied in glaucoma (Glen et al., 2014) and neuro-ophthalmic case findings (Szatmáry et al., 2002). Consequently, approaches using eye-movements (Murray et al., 2009; Mazumdar et al., 2014; Leitner et al., 2021b; Mao et al., 2021; Soans et al., 2021; Tatham et al., 2021) including tablet-based (Jones et al., 2019, 2021) and VR perimetry techniques (Deiner et al., 2020; Leitner and Hawelka, 2021; Leitner et al., 2021a; Narang et al., 2021; Razeghinejad et al., 2021) have been developed.

Our results show that participants overwhelmingly preferred the FOVE compared to the Tobii eye-tracker and the conventional SAP (statistically significant results were observed for all dimensions of the UEQ except for *Perspicuity* – see Table 5). Although the patient group was expected to rate the *Perspicuity* dimension high for SAP as they have prior experience in performing the test, it was encouraging to see that the control group naïve to all modalities reported high scores in terms of understanding the test instructions well (hence, no significant difference in *Perspicuity*). Since the Friedman’s test is essentially an omnibus test, we wanted to look further into which modalities, in particular, differed from each other. Therefore, *post hoc* analyses using Wilcoxon signed-rank tests were performed. In three of the five dimensions (*Competence*, *Immersion*, and *Aesthetics*), statistically significant differences (corrected for multiple comparisons in each dimension) were seen between the VR framework and the conventional SAP in all the clinical groups (see Supplementary Table 4). Interestingly, no significant differences were found for the *Comfort* dimension in the Friedman’s test for the neuro-ophthalmic group even though the significant differences were observed for the other two groups. The *post hoc* analysis, however, does not show significant differences for this dimension. Overall, however, participants clearly prefer the VR device (see *Attractiveness* dimension – Figure 6). This is in line with studies that have shown that SAP has been quite unpopular among patient groups (Gardiner and Demirel, 2008; Chew et al., 2016; McTrusty et al., 2017). These findings are significant as it gives credibility to the FOVE as an effective and enjoyable screening tool.

### Limitations and Future Directions

There are some limitations to our present study. We had fewer participants than the number of STP (45 participants vs. 80 STP). Therefore, we had to resort to dimensionality reduction and subsequent unsupervised clustering approaches instead of a more direct supervised learning technique. Another limitation is that the random walks of the luminance blob are currently restricted in its spatial range (±15° horizontal and vertical) even though the FOVE supports up to 100° field-of-view. This was done to keep a fair comparison between the approaches in the VR device and the screen-based eye tracker – the latter having an eye-tracking range of only 35° owing to its hardware structure. Although our current results indicate that the present spatial range is sufficient to form coherent clusters of patients and controls, it is quite possible that many individuals – for example, patients with PVFL having a localized scotoma in the far periphery will be missed.

Therefore, future directions should include several improvements that can be built based on the present work. Besides extending the spatial ranges of the stimuli to cover most of the visual field, it would also be helpful to look into STP patterns for homogenous types of VFD such that clinically useful field charts can be generated for the ophthalmologist. At present, we cannot yet make a direct comparison between the STP of a participant and the visual field chart obtained from SAP (which is why SAP is compared only in terms of UX in our study). Another area of active research is to make use of real-time head-tracking and pupillometry to monitor attention and malingering participants (Henson and Emuh, 2010) – akin to the video eye-monitoring feature of the HFA.

## Conclusion

We showed that patients can be screened for an underlying glaucomatous or neuro-ophthalmic VFD based on continuous EM tracking in a VR-based framework. The STP obtained from the VR-based framework can distinguish the participants according to their oculomotor characteristics. Furthermore, we showed that the STP estimated based on data gathered in the VR device is comparable to those estimated based using the screen-based eyetracker. In addition, participants from all the groups found the VR screening test to be the most *attractive*. We conclude that our EM-based approach implemented in VR will result in a useful, user-friendly, and portable test that can complement existing perimetric techniques in ophthalmic clinics.

## Data Availability Statement

The raw data supporting the conclusions of this article will be made available by the authors, without undue reservation.

## Ethics Statement

The studies human participants were reviewed and approved by the Ethics Board of All India Institute of Medical Sciences – Delhi (AIIMS) and the Indian Institute of Technology – Delhi (IITD). The patients/participants provided their written informed consent to participate in this study.

## Author Contributions

RSo, RR, and RSa designed the experiment. RSo, JJ, AB, and DR collected the data. RSo, RR, TG, and FC wrote the manuscript. RSa, RT, TG, and FC wrote the grants. All authors reviewed the manuscript.

## Conflict of Interest

RR is listed as an inventor on the European patent application “Grillini, A., Hernández-García, A., and Renken, J. R. (2019). Method, system and computer program product for mapping a visual field. EP19209204.7” filed by the UMCG. The patent application is partially based on some elements of the continuous tracking method described in this manuscript. The remaining authors declare that the research was conducted in the absence of any commercial or financial relationships that could be construed as a potential conflict of interest.

## Publisher’s Note

All claims expressed in this article are solely those of the authors and do not necessarily represent those of their affiliated organizations, or those of the publisher, the editors and the reviewers. Any product that may be evaluated in this article, or claim that may be made by its manufacturer, is not guaranteed or endorsed by the publisher.
