# Supplementary material for: Patients Prefer a Virtual Reality Approach Over a Similarly Performing Screen-Based Approach for Continuous Oculomotor-Based Screening of Glaucomatous and Neuro-Ophthalmological Visual Field Defects
Source: Front Neurosci. 2021 Oct 6;15:745355. doi: 10.3389/fnins.2021.745355 (PMC8526798; doi:10.3389/fnins.2021.745355)
Supplement: Supplementary file 1 [file Data_Sheet_1.pdf]

Patients prefer a virtual reality approach over a similarly performing screen-based approach for continuous oculomotor-based screening of glaucomatous and neuro-ophthalmological visual field defects

Rijul Saurabh Soans, Remco J. Renken, James John, Amit Bhongade, Dharam Raj, Rohit Saxena, Radhika Tandon, Tapan Kumar Gandhi, Frans W. Cornelissen

Supplementary Material

1 Procedure for filtering blinks in the eye-tracking data

1) The eye positions across the horizontal and vertical components are differentiated to obtain the respective gaze velocities.

2) In the time series of the vertical gaze velocities, all the spikes that go higher than a chosen threshold followed by a flat line or missing data are marked. The flat line refers to the first derivative of the vertical gaze velocity being zero. The rationale behind this procedure is due to the fact that video-based eye trackers wrongly interpret eye blinks as the pupil shifting suddenly upwards as the eyelids close. The gaze velocity thresholds for the VR device and the screen-based eyetracker are 60°/sec and 190°/sec, respectively.

3) Next, the beginning and the end of the blink period is delineated by recording the last valid position and subsequently dilating the blink period by five samples before and after the period.

4) Finally, this portion of missing data is filled with estimates that are inferred from forward and reverse autoregressive fits[1]. We use 10 samples preceding and succeeding the previously defined blink period for the autoregressive fits.

2 Supplementary Tables

Table 1. Visual field defects observed in the Neuro-Ophthalmology category

**Visual Field Defects**	**No. of eyes (n = 30)**
Enlargement of blind spot	6
Within normal limits	6
Hemianopia	5
Altitudinal	5
Generalized constriction	4

Paracentral scotoma	1
Peripheral scotomas	1
Quadrantanopia	1
Biarcuate	1

Table 2. Visual field defects observed in the Glaucoma category

**Visual Field Defects**	**No. of eyes (n = 29)**
Within normal limits	12
Biarcuate	5
Arcuate	4
Generalized constriction with	3
Paracentral scotoma	2
Inferior constriction	2
Superior constriction	1

Table 3. List of Spatio-Temporal Properties (STP) along with a description and the range of values

**Category**	**Property Name**	**Description**	**Range**
Spatial	1. Positional Error Distribution (PED): Amplitude	Describes the most frequent positional error observed. Higher values of amplitude for a mean of zero indicates better performance.	[0 1]
	2. Positional Error Distribution (PED): Mean	Describes the spatial offset. Values (in visual degrees) closer to zero indicate better performance.	[0 ∞]
	3. Positional Error Distribution (PED): Standard Deviation	Describes the spatial uncertainty: the spread of the positional deviations. Lower values indicate better performance.	[0 ∞]
	4. Positional Error Distribution (PED): Adjusted R-squared	Describes how close the positional error distribution resembles a Gaussian distribution. Values closer to 1 indicate better performance.	[-∞ 1]
	5. Average Velocity Cross-Correlogram (CCG): Amplitude	Shows the maximum correlation between the stimuli and gaze velocities. Higher values indicate better performance.	[-1 1]

Temporal	6. Average Velocity Cross-Correlogram (CCG): Mean	Describes the temporal lag between stimuli and gaze velocities (in ms). Lower values indicate better performance.	[0 ∞]
	7. Average Velocity Cross-Correlogram (CCG): Standard Deviation	Describes the temporal uncertainty: the time window (in ms) in which the observer is uncertain in their ability to track the stimulus. Lower values indicate better performance.	[0 ∞]
	8. Average Velocity Cross-Correlogram (CCG): Adjusted R-squared	Describes how close the temporal tracking performance resembles a Gaussian distribution. Values closer to 1 indicate better performance.	[-∞ 1]
Integrated	9. Observation noise variance	Describes the noise internal to the observer Sensory noise estimated by measuring the variance of the observational noise using a flipped Kalman filter. Lower values indicate better spatio-temporal performance.	[0 ∞]
	10. Similarity	Cosine similarity between gaze and stimulus vectors of positions. Higher values indicate better spatio-temporal performance.	[0 1]

Table 4. Post-hoc pairwise comparisons between the modalities for different patient groups. Each row tests the null hypothesis that the Sample 1 and the Sample 2 distributions are the same. Exact significances are displayed, and the significance level is 0.05. Statistically significant values after having been adjusted by the Bonferroni correction for multiple tests are highlighted.

Dimension	Participant Group	Sample 1 vs Sample 2	Test Statistic	Std. Error	Std. Test Statistic	Significance	Adjusted Significance
Competence	Controls	SAP vs Tobii	-0.429	0.309	-1.389	0.165	0.495
		**SAP vs FOVE**	**-0.857**	**0.309**	**-2.777**	**0.005**	**0.016**
		Tobii vs FOVE	-0.429	0.309	-1.389	0.165	0.495
	Glaucoma	SAP vs Tobii	-0.600	0.365	-1.643	0.1	0.301
		**SAP vs FOVE**	**-1.300**	**0.365**	**-3.560**	**<0.001**	**0.001**
		Tobii vs FOVE	-0.700	0.365	-1.917	0.055	0.166
	Neuro	SAP vs Tobii	-0.265	0.343	-0.772	0.440	1.000
		**SAP vs FOVE**	**-1.147**	**0.343**	**-3.344**	**0.001**	**0.002**
		**Tobii vs FOVE**	**-0.882**	**0.343**	**-2.572**	**0.010**	**0.030**
Perspicuity	Controls	SAP vs Tobii	N/A	N/A	N/A	N/A	N/A
		SAP vs FOVE	N/A	N/A	N/A	N/A	N/A
		Tobii vs FOVE	N/A	N/A	N/A	N/A	N/A
	Glaucoma	SAP vs Tobii	N/A	N/A	N/A	N/A	N/A
		SAP vs FOVE	N/A	N/A	N/A	N/A	N/A
		Tobii vs FOVE	N/A	N/A	N/A	N/A	N/A
	Neuro	SAP vs Tobii	N/A	N/A	N/A	N/A	N/A
		SAP vs FOVE	N/A	N/A	N/A	N/A	N/A
		Tobii vs FOVE	N/A	N/A	N/A	N/A	N/A
Immersion	Controls	SAP vs Tobii	-0.619	0.309	-2.006	0.045	0.135
		**SAP vs FOVE**	**-1.238**	**0.309**	**-4.012**	**<0.001**	**<0.001**
		Tobii vs FOVE	-0.619	0.309	-2.006	0.045	0.135
	Glaucoma	SAP vs Tobii	-0.600	0.365	-1.643	0.1	0.301
		**SAP vs FOVE**	**-1.300**	**0.365**	**-3.560**	**<0.001**	**0.001**
		Tobii vs FOVE	-0.700	0.365	-1.917	0.055	0.166
	Neuro	SAP vs Tobii	-0.206	0.343	-0.6	0.548	1.000
		**SAP vs FOVE**	**-1.029**	**0.343**	**-3.001**	**0.003**	**0.008**
		**Tobii vs FOVE**	**-0.824**	**0.343**	**-2.401**	**0.016**	**0.049**

Comfort	Controls	SAP vs Tobii	-0.333	0.309	-1.080	0.280	0.840
		SAP vs FOVE	-0.667	0.309	-2.160	0.031	0.092
		Tobii vs FOVE	-0.333	0.309	-1.080	0.280	0.840
	Glaucoma	SAP vs Tobii	-0.200	0.365	-0.548	0.584	1.000
		SAP vs FOVE	-0.500	0.365	-1.369	0.171	0.513
		Tobii vs FOVE	-0.300	0.365	-0.822	0.411	1.000
	Neuro	SAP vs Tobii	N/A	N/A	N/A	N/A	N/A
		SAP vs FOVE	N/A	N/A	N/A	N/A	N/A
		Tobii vs FOVE	N/A	N/A	N/A	N/A	N/A
Aesthetics	Controls	SAP vs Tobii	-0.286	0.309	-0.926	0.355	1.000
		**SAP vs FOVE**	**-0.786**	**0.309**	**-2.546**	**0.011**	**0.033**
		Tobii vs FOVE	-0.500	0.309	-1.620	0.105	0.316
	Glaucoma	SAP vs Tobii	-0.400	0.365	-0.1095	0.273	0.820
		**SAP vs FOVE**	**-0.900**	**0.365**	**-2.465**	**0.014**	**0.041**
		Tobii vs FOVE	-0.500	0.365	-1.369	0.171	0.513
	Neuro	SAP vs Tobii	-0.412	0.343	-1.200	0.230	0.690
		**SAP vs FOVE**	**-1.000**	**0.343**	**-2.915**	**0.004**	**0.011**
		Tobii vs FOVE	-0.588	0.343	-1.715	0.086	0.259

Table 5. Group means and corresponding 95% confidence intervals of three key STPs

				**Spatio-Temporal Feature**		
**Participant Group**	**Experiment Mode**	**Gaze Components**	**Measure**	**Temporal Lag**	**Temporal Uncertainty**	**Spatial Uncertainty**
**Neuro-Ophthalmology**	*Smooth*	X	Mean	0.2974	0.29	5.7528°
			CI	[0.2745, 0.3204]	[0.2643, 0.3158]	[5.1404°, 6.3652°]
		Y	Mean	0.3076	0.2779	4.5074°
			CI	[0.2841, 0.3310]	[0.2534, 0.3025]	[4.0232°, 4.9917°]
	Displaced	X	Mean	0.3162	0.2293	6.6184°
			CI	[0.2869, 0.3456]	[0.2162, 0.2425]	[5.7664°, 7.4704°]
		Y	Mean	0.3377	0.2296	5.066°
			CI	[0.3064, 0.3690]	[0.2098, 0.2494]	[4.4276°, 5.7056°]
**Glaucoma**	*Smooth*	X	Mean	0.1730	0.2403	3.9385°
			CI	[0.1390, 0.2071]	[0.2232, 0.2574]	[3.6085°, 4.2686°]
		Y	Mean	0.2240	0.2141	3.7505°
			CI	[0.2049, 0.2431]	[0.1982, 0.23]	[3.3471°, 4.1540°]
	Displaced	X	Mean	0.3402	0.2810	6.1402°
			CI	[0.3012, 0.3792]	[0.2517, 0.3104]	[5.2101°, 7.0703°]
		Y	Mean	0.3617	0.2741	5.5883°
			CI	[0.3183, 0.4051]	[0.2422, 0.306]	[4.7655°, 6.411°]
	Smooth	X	Mean	0.1570	0.2161	2.7678°
			CI	[0.1486, 0.1653]	[0.2081, 0.2241]	[2.4346°, 3.1010°]
			Mean	0.1760	0.1844	2.4380°

**Controls**		Y	CI	[0.1684, 0.1836]	[0.1796, 0.1892]	[2.2709°, 2.6051°]
	Displaced	X	Mean	0.2014	0.2014	3.3307°
			CI	[0.1916, 0.2111]	[0.1952, 0.2076]	[3.0228°, 3.6385°]
		Y	Mean	0.2104	0.1879	3.0923°
			CI	[0.2025, 0.2183]	[0.1779, 0.198]	[2.881°, 3.3037°]

Table 6. List of all the 80 STPs in descending order of their pairwise correlation coefficient between the VR and the eye tracker setups. Fifty-three features out of the total 80 STPs were found to have significant correlation.

SI. No.	STP	Pairwise correlation coefficient	Statistical Significance
1	LeftEye_SmoothMode_Horizontal_CCG_AdjR^2	0.9749	Yes, p<0.05
2	LeftEye_DisplacedMode_Horizontal_PED_StdDev	0.9175	Yes, p<0.05
3	LeftEye_DisplacedMode_Vertical_PED_StdDev	0.8894	Yes, p<0.05
4	RightEye_SmoothMode_Horizontal_CCG_AdjR2	0.8550	Yes, p<0.05
5	LeftEye_DisplacedMode_Vertical_CCG_StdDev	0.8265	Yes, p<0.05
6	RightEye_SmoothMode_Horizontal_PED_StdDev	0.8164	Yes, p<0.05
7	LeftEye_SmoothMode_Horizontal_PED_StdDev	0.7815	Yes, p<0.05
8	LeftEye_DisplacedMode_Horizontal_CCG_Mean	0.7576	Yes, p<0.05
9	LeftEye_SmoothMode_Vertical_CCG_Mean	0.7149	Yes, p<0.05
10	LeftEye_DisplacedMode_Vertical_CCG_Mean	0.7056	Yes, p<0.05
11	LeftEye_DisplacedMode_Horizontal_CCG_AdjR^2	0.6974	Yes, p<0.05
12	LeftEye_DisplacedMode_Horizontal_KalmanR	0.6299	Yes, p<0.05
13	LeftEye_DisplacedMode_Horizontal_Similarity	0.6250	Yes, p<0.05
14	RightEye_DisplacedMode_Horizontal_CCG_AdjR^2	0.6206	Yes, p<0.05
15	LeftEye_SmoothMode_Vertical_CCG_AdjR^2	0.6094	Yes, p<0.05
16	RightEye_DisplacedMode_Horizontal_Similarity	0.5908	Yes, p<0.05
17	LeftEye_SmoothMode_Vertical_PED_StdDev	0.5757	Yes, p<0.05
18	LeftEye_DisplacedMode_Vertical_Similarity	0.5705	Yes, p<0.05
19	LeftEye_SmoothMode_Horizontal_PED_AdjR^2	0.5673	Yes, p<0.05
20	LeftEye_DisplacedMode_Horizontal_CCG_Amplitude	0.5639	Yes, p<0.05
21	RightEye_DisplacedMode_Horizontal_CCG_Amplitude	0.5529	Yes, p<0.05
22	LeftEye_SmoothMode_Horizontal_CCG_Mean	0.5469	Yes, p<0.05
23	LeftEye_SmoothMode_Horizontal_Similarity	0.5364	Yes, p<0.05
24	LeftEye_DisplacedMode_Vertical_CCG_AdjR^2	0.5245	Yes, p<0.05
25	LeftEye_DisplacedMode_Horizontal_CCG_StdDev	0.5176	Yes, p<0.05
26	LeftEye_DisplacedMode_Vertical_KalmanR	0.4988	Yes, p<0.05
27	LeftEye_DisplacedMode_Vertical_PED_AdjR^2	0.4926	Yes, p<0.05
28	RightEye_SmoothMode_Vertical_CCG_Mean	0.4886	Yes, p<0.05
29	RightEye_SmoothMode_Vertical_CCG_Amplitude	0.4828	Yes, p<0.05
30	RightEye_DisplacedMode_Horizontal_CCG_StdDev	0.4547	Yes, p<0.05
31	RightEye_DisplacedMode_Vertical_CCG_Mean	0.4539	Yes, p<0.05
32	RightEye_SmoothMode_Vertical_CCG_AdjR^2	0.4407	Yes, p<0.05
33	LeftEye_SmoothMode_Horizontal_KalmanR	0.4375	Yes, p<0.05
34	RightEye_DisplacedMode_Vertical_CCG_AdjR^2	0.4366	Yes, p<0.05
35	RightEye_DisplacedMode_Vertical_CCG_Amplitude	0.4287	Yes, p<0.05
36	RightEye_DisplacedMode_Vertical_CCG_StdDev	0.4157	Yes, p<0.05
37	RightEye_DisplacedMode_Vertical_PED_StdDev	0.4116	Yes, p<0.05
38	RightEye_DisplacedMode_Horizontal_CCG_Mean	0.4048	Yes, p<0.05
39	LeftEye_SmoothMode_Horizontal_CCG_StdDev	0.3977	Yes, p<0.05
40	RightEye_SmoothMode_Horizontal_CCG_Mean	0.3972	Yes, p<0.05
41	RightEye_DisplacedMode_Vertical_Similarity	0.3928	Yes, p<0.05
42	LeftEye_DisplacedMode_Horizontal_PED_AdjR^2	0.3898	Yes, p<0.05
43	LeftEye_SmoothMode_Vertical_CCG_Amplitude	0.3771	Yes, p<0.05
44	RightEye_DisplacedMode_Horizontal_PED_StdDev	0.3734	Yes, p<0.05
45	LeftEye_SmoothMode_Horizontal_CCG_Amplitude	0.3701	Yes, p<0.05
46	LeftEye_SmoothMode_Vertical_CCG_StdDev	0.3531	Yes, p<0.05
47	RightEye_SmoothMode_Horizontal_CCG_Amplitude	0.3522	Yes, p<0.05
48	RightEye_SmoothMode_Vertical_PED_StdDev	0.3513	Yes, p<0.05
49	LeftEye_SmoothMode_Vertical_KalmanR	0.3314	Yes, p<0.05

50	RightEye_SmoothMode_Horizontal_Similarity	0.3300	Yes, p<0.05
51	RightEye_DisplacedMode_Horizontal_KalmanR	0.3272	Yes, p<0.05
52	RightEye_SmoothMode_Horizontal_CCG_StdDev	0.3158	Yes, p<0.05
53	RightEye_SmoothMode_Vertical_CCG_StdDev	0.2976	Yes, p<0.05
54	LeftEye_DisplacedMode_Horizontal_PED_Amplitude	0.2932	No, p=0.0506
55	RightEye_DisplacedMode_Horizontal_PED_AdjR^2	0.2925	No, p=0.0512
56	LeftEye_SmoothMode_Horizontal_PED_Mean	0.2860	No, p=0.0568
57	RightEye_DisplacedMode_Horizontal_PED_Amplitude	0.2837	No, p=0.0590
58	RightEye_SmoothMode_Horizontal_PED_Amplitude	0.2807	No, p=0.0618
59	RightEye_SmoothMode_Vertical_Similarity	0.2799	No, p=0.0626
60	LeftEye_SmoothMode_Horizontal_PED_Amplitude	0.2769	No, p=0.0656
61	LeftEye_SmoothMode_Vertical_PED_AdjR^2	0.2684	No, p=0.0746
62	LeftEye_SmoothMode_Vertical_Similarity	0.2394	No, p=0.1132
63	RightEye_SmoothMode_Vertical_PED_Amplitude	0.2342	No, p=0.1214
64	RightEye_SmoothMode_Horizontal_PED_AdjR^2	0.2334	No, p=0.1229
65	LeftEye_DisplacedMode_Vertical_PED_Mean	0.2324	No, p=0.1244
66	LeftEye_DisplacedMode_Vertical_PED_Amplitude	0.2228	No, p=0.1412
67	LeftEye_DisplacedMode_Vertical_CCG_Amplitude	0.1918	No, p=0.2068
68	RightEye_DisplacedMode_Vertical_KalmanR	0.1827	No, p=0.2296
69	RightEye_SmoothMode_Vertical_PED_AdjR^2	0.1368	No, p=0.3703
70	RightEye_SmoothMode_Vertical_KalmanR	0.1337	No, p=0.3813
71	RightEye_SmoothMode_Horizontal_KalmanR	0.1221	No, p=0.4243
72	LeftEye_SmoothMode_Vertical_PED_Amplitude	0.1173	No, p=0.4427
73	RightEye_DisplacedMode_Vertical_PED_AdjR^2	0.0809	No, p=0.5975
74	RightEye_SmoothMode_Vertical_PED_Mean	0.0504	No, p=0.7425
75	RightEye_DisplacedMode_Horizontal_PED_Mean	0.0238	No, p=0.8767
76	RightEye_SmoothMode_Horizontal_PED_Mean	-0.0067	No, p=0.9652
77	RightEye_DisplacedMode_Vertical_PED_Mean	-0.0112	No, p=0.9420
78	RightEye_DisplacedMode_Vertical_PED_Amplitude	-0.0476	No, p=0.7561
79	LeftEye_SmoothMode_Vertical_PED_Mean	-0.1469	No, p=0.3355
80	LeftEye_DisplacedMode_Horizontal_PED_Mean	-0.2217	No, p=0.1432

3 Supplementary Figure

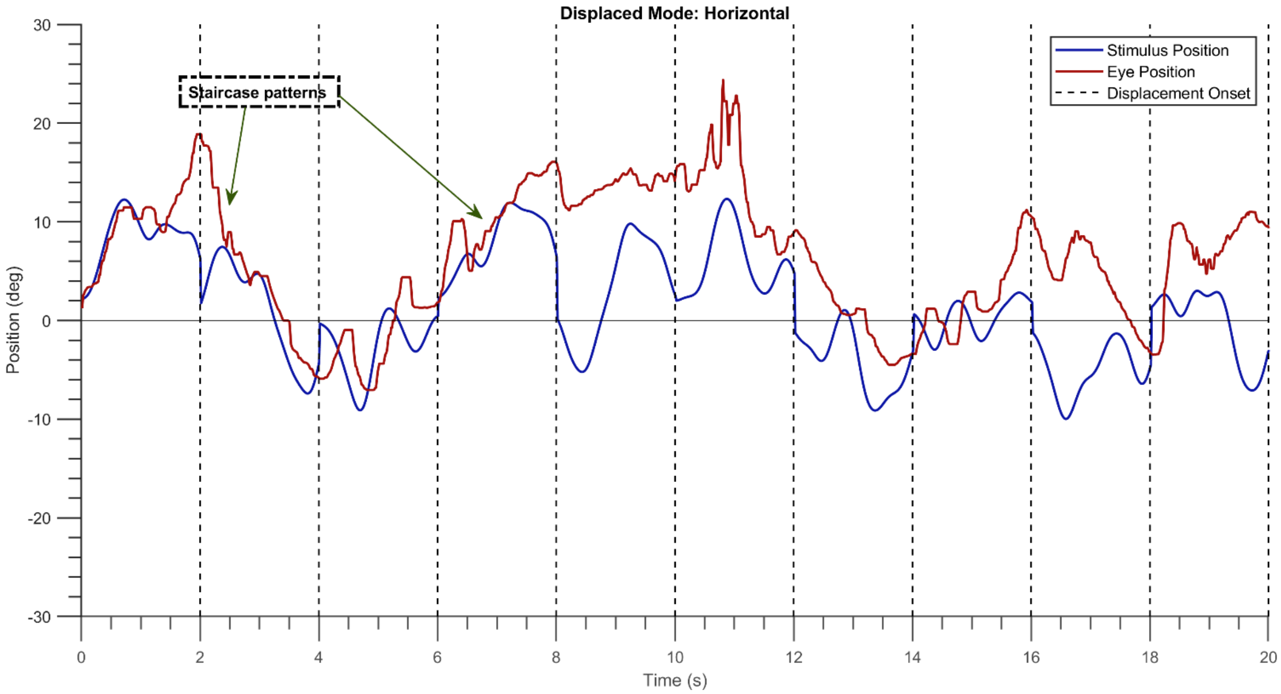

Figure 1. Eye movement patterns made by a hemianopic patient in a "displaced" mode trial. Staircase movement of the gaze is observed usually just before and after the luminance blob makes a jump. This

seems to be a predictive visual search strategy by the participant – as explained by Meienberg et al.,[2] in hemianopic patients - as the participant expects the target to jump to a new random location every 2 seconds. This behavior may have altered the STP of EM in the neuro-ophthalmic group (in particular, contributing to lower temporal uncertainties while still having higher lags and spatial uncertainty values)

4 Supplementary References

1. Kay SM. *Modern Spectral Estimation: Theory and Application*. Englewood Cliffs, NJ, USA: Prentice Hall; 1988.

2. Meienberg O, Zangemeister WH, Rosenberg M, Hoyt WF, Stark L. Saccadic eye movement strategies in patients with homonymous hemianopia. *Ann Neurol*. 1981;9(6):537-544. doi:10.1002/ana.410090605
